# Ion Channels in Native Chloroplast Membranes: Challenges and Potential for Direct Patch-Clamp Studies

**DOI:** 10.3389/fphys.2015.00396

**Published:** 2015-12-22

**Authors:** Igor Pottosin, Oxana Dobrovinskaya

**Affiliations:** Centro Universitario de Investigaciones Biomédicas, Universidad de ColimaColima, Mexico

**Keywords:** chloroplast envelope, cation channel, ClC channel, magnesium, patch-clamp, porin, proton-motive force, thylakoid

## Abstract

Photosynthesis without any doubt depends on the activity of the chloroplast ion channels. The thylakoid ion channels participate in the fine partitioning of the light-generated proton-motive force (p.m.f.). By regulating, therefore, luminal pH, they affect the linear electron flow and non-photochemical quenching. Stromal ion homeostasis and signaling, on the other hand, depend on the activity of both thylakoid and envelope ion channels. Experimentally, intact chloroplasts and swollen thylakoids were proven to be suitable for direct measurements of the ion channels activity via conventional patch-clamp technique; yet, such studies became infrequent, although their potential is far from being exhausted. In this paper we wish to summarize existing challenges for direct patch-clamping of native chloroplast membranes as well as present available results on the activity of thylakoid Cl^−^ (ClC?) and divalent cation-permeable channels, along with their tentative roles in the p.m.f. partitioning, volume regulation, and stromal Ca^2+^ and Mg^2+^ dynamics. Patch-clamping of the intact envelope revealed both large-conductance porin-like channels, likely located in the outer envelope membrane and smaller conductance channels, more compatible with the inner envelope location. Possible equivalent model for the sandwich-like arrangement of the two envelope membranes within the patch electrode will be discussed, along with peculiar properties of the fast-activated cation channel in the context of the stromal pH control.

## Introduction

Chloroplasts originated from endosymbiosis of an ancestral cyanobacterium and a primitive eukaryotic cell. The two envelope membranes, outer (OE), and inner (IE) ones are homologous to external and plasma membranes of Gram-negative bacteria, which is confirmed by the presence of galactolipids and β-barrel proteins (porins) in the OE and external membrane of free-living Gram-negative bacteria (Inoue, 2007; Gould et al., 2008; Breuers et al., 2011). The two envelope membranes are aligned close to each other, separated by only 1–2 membrane thickness (5–10 nm as compared to a typical chloroplast size of 3–4 μm); IE and OE come to even closer proximity at contact sites (Inoue, 2007). Membrane/compartment arrangements in chloroplasts are different from those in mitochondria. Whereas, the outer mitochondrial membrane may be compared with the OE, the inner mitochondrial membrane combines both energy-coupling and metabolite exchange functions. As the two mitochondrial membranes are mostly separated, the activity of outer and inner membrane channels could be directly studied by patch-clamp technique, using intact mitochondria or swollen mitoplasts, respectively (Szabo and Zoratti, 2014). Double-membrane bound chloroplasts represent technically a more challenging task, as will be discussed in this paper. The stroma of chloroplasts, however, may be compared with the mitochondrial matrix: it is a slightly alkaline (compared to the cytosol) compartment with a high biosynthetic potential. Nine out of twenty essential amine acids are synthesized exclusively in the stroma, as well as are fatty acids, carbohydrates and triose phosphates, NADPH, purines, and a variety of secondary metabolites (Breuers et al., 2011; Rolland et al., 2012). The inner envelope contains a variety of solute transporters, mediating export of photoassimilates and import of substrates, as well as ion exchange (Weber and Linka, 2011). Both functions can be complemented by the activity of inner envelope ion channels. In addition, as it will be discussed in this review, cation channels, a putative H^+^-ATPase, and monovalent cation/H^+^ exchangers of the IE could assist maintenance of metabolically optimal alkaline pH in the stroma and control chloroplast volume. In the OE, transport activity of porin-like channels appears to dominate in both ion and metabolite traffic (Duy et al., 2007) and their differential substrate selectivity and regulation will be discussed.

**Figure 1:**
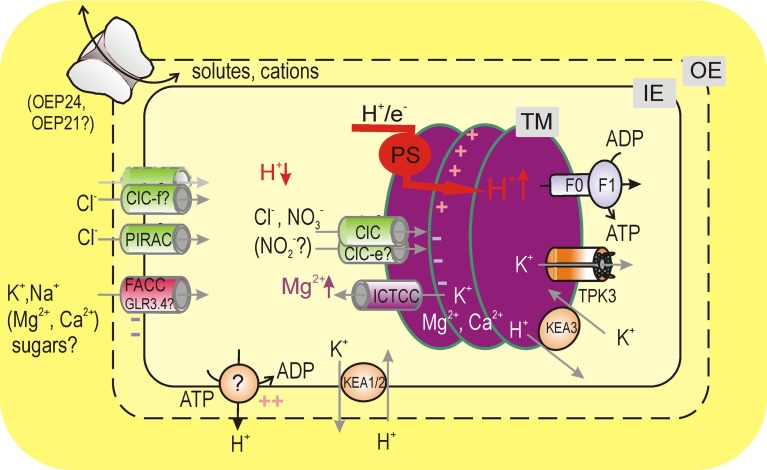
**Chloroplast ion transport under the light**. Light-driven export of H^+^ into the thylakoid lumen by photosynthetic electron transfer chain (PS) causes a hyperpolarization of the thylakoid ΔΨ. At steady state, this voltage difference is partly dissipated by channel-mediated fluxes of anions, K^+^, and Mg^2+^. Light-driven H^+^ and parallel Cl^−^ fluxes to the thylakoid lumen cause the depletion of these ions in stroma, which is compensated by their uptake across the envelope. For maintenance of alkaline stromal pH, H^+^ could be actively extruded to cytosol by the IE H^+^ pump, which requires a counter influx of monovalent cations across the envelope for electrical balance. K^+^/H^+^ exchange across the envelope is essential for control of the chloroplast volume and stromal pH. Abbreviations: TM, IE, and OE are thylakoid, inner envelope, and outer envelope membranes, F0F1 is thylakoid ATP-synthetase (F-type H^+^-ATPAse), TPK3 (tandem-pore K^+^ 3 channel, functionally characterized in recombinant system). *In situ* functionally (by patch-clamp) detected channels were: ClC (anion-selective channel from a ClC family), ICTCC (intermediate-conductance thylakoid cation channel), FACC (fast activating chloroplast cation channel), PIRAC (protein import related anion channel), and outer envelope porins (most possibly, active OEP24 or OEP21). Other: GLR3.4 (glutamate receptor type 3.4 channel) and KEA1/2 (cation/proton antiporters from family 2, CPA2). Another member of the CPA2 family, the thylakoid-localized KEA3, accelerates dissipation of the transthylakoid ΔpH upon the light offset.

The thylakoid membrane is an internal membrane of the chloroplast, representing a complex network of grana stacks connected by stromal lamellae (for thylakoid structure, see recent review by Pribil et al., 2014). The thylakoid membrane, being a site for light-driven photosynthetic reactions, harbors photosynthetic pigments and protein complexes of the electron transfer chain as well as F-type H^+^-ATPase, which performs photophosphorylation. In variance to mitochondria, chloroplasts store a proton-motive force (p.m.f.), which fuels the ATP-synthesis, mainly as ΔpH rather than the transmembrane electric potential difference (ΔΨ). Thus, thylakoid lumen represents a unique acidic compartment. Interconversion between ΔpH and ΔΨ across the thylakoid membrane is under environmental control and the steady state p.m.f. partitioning critically depends on the activity of thylakoid ion channels (Kramer et al., 2004). Partial dissipation of ΔΨ, generated by light-driven H^+^-pumping into thylakoid lumen is achieved via passive fluxes of anions (Cl^−^), K^+^, and Mg^2+^ (Hind et al., 1974). Accordingly, activities of anion and nonselective cation channels were revealed by direct patch-clamping of native thylakoid membranes, whereas activity of thylakoid K^+^-selective channels was assayed in a reconstituted system (Figure 1). Specific functional properties of these channels will be discussed in the following.

This review is centered in chloroplast ion channels, which could be directly measured by patch-clamp technique. For a broader overview of the chloroplast ion transport system in the physiological context an interested reader could consult a recent review by Pottosin and Shabala (2015).

## Ion transport across the thylakoid membrane

### Ion fluxes assist the conversion of electrical potential difference to ΔpH

The difference of electrochemical potential for H^+^ (ΔμH^+^), generated under light, is an obligatory intermediate for the photo-phosphorylation, ATP synthesis by H^+^-transporting F-type ATPase. Often, instead of (ΔμH^+^), the related parameter, proton-motive force (p.m.f.) is used, p.m.f. = ΔΨ-ZΔpH (Mitchell, 1966), where Z is approximately 59 mV at room temperature, ΔΨ and ΔpH represent the differences in electrical potential and pH across the thylakoid membrane, respectively. Thermodynamically, electrical and chemical components of the p.m.f. are equivalent; however, measurements of turnover rates with isolated F-ATPases demonstrated that the mitochondrial and bacterial F-ATPases critically require the presence of a substantial ΔΨ, whereas chloroplast F-ATPase depends on the ΔΨ less critically (Fischer and Gräber, 1999; Cruz et al., 2001). It was considered for a long time that under a steady state light the p.m.f. in chloroplasts consists almost completely of ΔpH, whereas the steady state ΔΨ is negligibly small (~−10 mV stroma negative) (e.g., Bulychev et al., 1972; Remiš et al., 1986). *In vivo* studies, however, demonstrated that ΔΨ may yield up to 50% of p.m.f. or −60 mV (Cruz et al., 2001; Kramer et al., 2003, 2004; Klughammer et al., 2013). Thus, ΔpH, which yields at least a half of the p.m.f., is about 1–1.5 pH units under light. Assuming a constant stromal pH of 8 under light (see the section on stromal pH control), this yields pH 6.5–7 in the lumen. Under extreme conditions (strong light, low CO_2_), luminal pH may drop to 6–5.5 (Tikhonov et al., 1981; Schönknecht et al., 1995; Takizawa et al., 2007). On one side, an acidic pH in the lumen is necessary for the stimulation of non-photochemical quenching, which prevents the photodamage of the reaction center of the Photosytem II (PSII) and reactive oxygen species (ROS) generation at excessive light. On the other side, a luminal pH below 6.5 strongly reduced the linear electron transport flow from the cytochrome b6f complex to the reaction center of Photosystem I (PSI), and a pH below 6 may even cause a loss of function of the water-splitting complex (exclusive electron donor for the PSII) and of the plastocyanin, electron carrier between b6f and Photosystem I, due to the replacement of the functionally important Ca^2+^ and Cu in these proteins by protons (Kramer et al., 2003). Obviously, partitioning of p.m.f. into ΔΨ and ΔpH should be finely tuned. Existence of a (stroma) negative steady-state ΔΨ under light implies a driving force for anion efflux from and cation influx to the stroma. If the thylakoid membrane conductance for physiologically abundant cations or anions were large, ΔΨ will collapse, and excessive lumen acidification will result. As it did not happen, one has to assume that the functional expression of respective ion channels, their selective permeability, the availability of transported ions, and the control of channels' open probability by physiologically relevant factors (membrane voltage, pH, Ca^2+^, etc.) should be set exactly to balance the light-driven H^+^ pump-generated current at *given* ΔΨ *and pH* in stroma and lumen. Current, generated by the light-driven H^+^ pump (photosynthetic electron transfer chain), was directly evaluated by patch-clamp technique and could reach up to tens of pA for a single chloroplast under strong light (Bulychev et al., 1992; Muñiz et al., 1995). Above considerations emphasize the importance of *quantitative* measurements of the activity of thylakoid ion channels *in situ*, under conditions close to the physiological ones.

### Patching of thylakoids is technically challenging but feasible

First patch-clamp recording on the photosynthetic membrane was achieved by Schönknecht et al. (1988), who employed hypotonic shock to inflate the thylakoid compartment. Chloroplasts, poised into the hypotonic medium (isotonic saline, diluted 4–5 times with pure water) rapidly broke down, releasing large (10–40 μm in diameter) transparent vesicles (blebs), which are swollen thylakoids. Such a large size of blebs is a consequence of the interconnection of all thylakoids within the network, which encloses therefore a common lumen (Schönknecht et al., 1990; Shimoni et al., 2005). Thylakoid blebs conserved light-induced membrane polarization, photochemical activity, and are able to photophosphorylate (Campo and Tedeschi, 1985; Allen and Holmes, 1986; Hinnah and Wagner, 1998). Forming of a high-quality gigaOhm seal between glass microelectrode and bleb represented, however, a major problem, which may be at least partially caused by a very low free lipid and high protein content of the thylakoid membrane. Together with another energy-coupling membrane, the inner mitochondrial one, thylakoids display the highest protein to lipid ratio of 3.4:1 (w/w); lipids cover only 20% of the membrane surface and 60% of total lipid is immobilized within the first protein solvation shell (Kirchhoff et al., 2002; Kirchhoff, 2008). In comparison, the outer and inner chloroplast envelope membranes present a protein to lipid w/w ratio of 0.35:1 and 1.1:1, respectively (Inoue, 2007). Abundance of integral proteins, protruding far from the bilayer (e.g., F-type H^+^-ATPase), which have to denaturate against the glass, to allow direct contact between glass and lipid, would at least make the overall sealing process longer (Suchyna et al., 2009). Yet, although the inner mitochondrial membrane displays the same high protein to lipid ratio, respective studies greatly outnumber those available so far for thylakoids (Schindl and Weghuber, 2012; Szabo and Zoratti, 2014). The difference in lipid composition of the two membranes may be an additional problem. The mitochondrial inner membrane, similar to the plasma membrane, endoplasmic reticulum, Golgi, and endosomal membranes, is rich in phospholipids, including a high percentage (about 20%) of negatively (2-) charged cardiolipin (diphosphatydyl glycerol), specific for mitochondria (van Meer et al., 2008). Contrary to this, the thylakoid membrane is mainly made of galactolipids (84%), with 7% of negatively charged sulfolipids and phosphatydylglycerol as a sole phospholipid (Block et al., 2007). The adhesion energy between glass and lipid bilayer varies by up to one order of magnitude as a function on the lipid composition (Ursell et al., 2011). This has been proven for different combinations of phospholipids, while respective data for galactolipids is missing. However, as gigaOhm seal formation is mainly stabilized by van der Waals forces (Suchyna et al., 2009), the presence of fixed dipoles in phospholipids as compared to glycolipids, would facilitate a tight seal formation in the first case. Although the first successful patch-clamp study was performed on a species with abnormally large (up to 40 μm in diameter) chloroplasts, *Peperomia metalica* (Schönknecht et al., 1988), bleb size does not appear to be a problem for tight sealing with a patch-pipette tip. Moreover, in our hands, blebs originated from *Peperomia* chloroplasts were proven to be more difficult to patch as compared to more typical chloroplasts of spinach. A critical moment was the time spent between a bleb formation in the experimental chamber and the attempt to obtain a tight seal, which should not exceed 10–15 min. (Pottosin and Schönknecht, 1995a, 1996; Hinnah and Wagner, 1998). Failure in fulfilling this condition resulted in the absence of stable tight seals (Enz et al., 1993). The presence of high divalent cation concentrations (e.g., 5 mM MgCl_2_) at both membrane sides was also mandatory. It should also be noted that albeit achievement of high (up to 10 GOhm) resistance seals between the patch pipette and a thylakoid bleb could be done routinely, obtained membrane patches were extremely fragile and rarely withstood voltages higher than 40 mV by absolute value. So far, any attempt to get access to the bleb interior (whole thylakoid configuration) by application of a short pulse of high voltage or of strong suction resulted in the loss of the sample in 100% of cases. Yet, a very promising perspective to gain low resistance access to the thylakoid lumen without loss of a tight seal may be patch perforation by incorporation of channel-forming antibiotics (e.g., gramicidin, Schönknecht et al., 1990) into the patch pipette tip.

An alternative for a direct patch-clamping of the intact thylakoid membrane could be a dilution of the thylakoid lipid, either by its fusion into azolectin liposomes (Enz et al., 1993) or by incorporation of thylakoid membranes or purified channel protein into artificial lipid bilayers (Tester and Blatt, 1989; Li et al., 1996; Carraretto et al., 2013). Yet, it should be noted that incorporation of any external material into the lipid bilayer could produce an artificial channel. This may not necessarily be a protein responsible for such activity, as defects caused by lipid peroxidation, detergents, and/or bacterial contamination could be equally problematic (Labarca and Latorre, 1992; Pelzer et al., 1993). Consequently, one needs to have additional criteria. Usage of specific channel agonists or antagonists should be a solution, but as far as we know such a test was not performed for thylakoid channels, and even reproducibility of channel characteristics may be considered a problem in this case. This is not surprising, as an artificial environment can alter the channels' function or even induce a channel-like behavior in proteins, which do not form channels under physiological conditions, as it is true, for instance, for the chloroplast triose phosphate/phosphate translocator or phosphate carrier of inner mitochondrial membrane (Schwarz et al., 1994; Herick et al., 1997).

### Anion-selective channel: evidence for a functional ClC in the thylakoid membrane

A 100 pS (in 100 mM KCl) voltage-dependent channel, with almost perfect selectivity for anions (Cl^−^, ${\text{NO}}_{3}^{-}$) over K^+^ was first time reported for intact thylakoid membrane from chloroplasts, isolated from leaves of *P. metalica* (Schönknecht et al., 1988). Later on, quite similar in their conductance (Figure 1), selectivity, and voltage-dependent kinetics channels were reported also for thylakoids of a Charophyte alga *Nitellopsis obtuse* and spinach (Pottosin and Schönknecht, 1995a,b). Notably, no such channel could be detected upon reconstitution of spinach thylakoid membranes into giant azolectin liposomes (Enz et al., 1993), which emphasizes the importance of studies on native membranes. Thylakoid anion channel by its conductance is reminiscent of the so-called “mitochondrial Centium picoSiemens” (mCS) anion channel of the inner membrane. Yet, mCS channel has a low Cl^−^/K^+^ selectivity, is inhibited by Mg^2+^, and has a distinct voltage dependence (Sorgato et al., 1987; Borecký et al., 1997; Tomaskova and Ondrias, 2010; Szabo and Zoratti, 2014) as compared to the thylakoid channel. The voltage dependence of the thylakoid anion channel was bell-shaped, with a maximal open probability at zero voltage and a steep decrease of it at both positive and negative potentials, so that at ±50 mV the steady open probability was close to zero (Pottosin and Schönknecht, 1995a, 1996). Increase of Cl^−^ concentration on the luminal side tended to increase the channel's open probability, which may serve as a positive feedback mechanism upon light-induced anion accumulation in thylakoids (Pottosin and Schönknecht, 1995a). It has been shown that the thylakoid anion channel voltage-dependence is controlled by the two gating processes: a rapid (ms) one, which tends to favor open probability at stroma-positive potential, and a slow (sub-seconds to seconds), which, in contrast, favors the channel opening at stroma-negative potentials. Consequently, changing the voltage polarity evoked transient channel openings, relaxing slower when the potential was switched from positive to negative values (Schönknecht et al., 1988; Pottosin and Schönknecht, 1995a). Change of the polarity of transthylakoid ΔΨ naturally occurs upon light-dark transitions, with the light onset or offset making stromal side more negative (Figure 1) or positive, respectively (Kramer et al., 2003). In this context, transient “on demand” activation of the anion channel may be especially important upon the light onset, when the channel-mediated current will accelerate the achievement of a steady state ΔΨ at the light. Often, thylakoid patches displayed the activity of multiple anion channels. Whereas, fast close-open transitions of these channels were independent each of other, a common slow gate was detected, which could close a pair of channels simultaneously, with a probability far above being merely a coincidence (Pottosin and Schönknecht, 1995a). Thus, thylakoid anion channels displayed a coupled (“double-barreled”) behavior, which is a hallmark characteristic for anion channels from the ClC family.

The ClC family is dominated by 2Cl^−^/1H^+^ antiporters, and only a small part of it displays channel-like behavior. Such “broken” transporters preserve the activation by external protonation and have some remnant permeability to H^+^, which drives their slow gating cycle out of equilibrium (Miller, 2006; Zifarelli and Pusch, 2010a,b). ClC channels and transporters, however, present only minor structural differences. A ClC protein possesses two homologous extended Cl^−^ pathways, which may be locked open in channels, resulting in long-living transmembrane pores. The presence of those two homomeric Cl^−^ pores, whose opening may be controlled by a common gate, serves as a natural explanation for a “double-barreled” behavior. In the ClC transporter structure, two glutamate residues, external, and internal ones, are essential for H^+^ sensing and transport, respectively. Consequently, channel members of ClC family lack the “H^+^ transport” glutamate, but preserve the external one, controlling the fast gate (Miller, 2006; Zifarelli and Pusch, 2010a). In *Arabidopsis*, of seven ClC members only two, AtClC-e and AtClC-f, are potentially channel-forming, according to this criterion (Zifarelli and Pusch, 2010a). A ClC-f product was localized in the outer chloroplast envelope and in Golgi membranes, whereas ClC-e is targeted at the thylakoid membrane (Teardo et al., 2005; Marmagne et al., 2007). One problem, however, is related to these tentative ion channels: they are lacking a highly conserved sequence, underlying Cl^−^ (GSGIP) or ${\text{NO}}_{3}^{-}$ (GPGIP) selectivity: in ClC-e and –f these are replaced by ESAGK and EILDQ, respectively. Thus, current state-of-art approaches may not predict to which selectivity this arrangement may account (Zifarelli and Pusch, 2010a). The knockout of either of these genes disturbed nitrate homeostasis, although it remains unclear, whether it was a consequence of the cessation of ${\text{NO}}_{3}^{-}$ and/or or ${\text{NO}}_{2}^{-}$ transport by these channels, because multiple parameters were affected, including decrease of nitrate uptake by roots and expression of high-affinity nitrate transporters, increased nitrite and decreased nitrate levels (Monachello et al., 2009).

Unanswered questions include before all an unambiguous identification of the thylakoid anion channel. If it is ClC-e, as proposed by us and by others (Checchetto et al., 2013; Pfeil et al., 2014), then a direct proof should be the presence or absence of anion channels activity in thylakoids of WT and knock-out plants, respectively. This reinforces the need of a direct patch-clamping of *Arabidopsis* thylakoids, the problem is technically not solved yet, but is definitely a rewarding one. Lack of ClC-e function plants did not show a clear phenotype (Marmagne et al., 2007), but p.m.f. partitioning was not addressed so far, which calls for a further study. We also believe that testing the pH regulation of the thylakoid anion channel activity is physiologically quite relevant, due to the large changes of luminal and stromal pH upon dark/light transitions and due to the existence of a strong regulation of ClC-type channels by protons. Finally, although up to now no specific blocker of ClC channels is known, non-specific blockers like 9-anthracene carboxylate, flufenamic or niflumic acid, and clofibric acid derivates are available (Zifarelli and Pusch, 2012) and should be tested directly against thylakoid anion channels, to reveal their potential for further usage in diverse functional assays (e.g., p.m.f. steady state partitioning).

## Divalent cation-permeable channels

Mg^2+^ influx from thylakoids into stroma is recognized as a part counterbalancing process for light-driven H^+^ transport into the lumen for a long time, although its contribution decreases upon the increase of external (cytosolic) K^+^ (Hind et al., 1974). However, light onset caused a very substantial increase of stromal Mg^2+^, from 0.5–1 to 2–3 mM (Portis and Heldt, 1976; Ishijima et al., 2003). Given relatively low envelope permeability for Mg^2+^ (Gimmler et al., 1974), this change is due to the mobilization of Mg^2+^ from thylakoids. It should be noted that as specific volumes of thylakoids to stroma are related as 1:15 (Heldt et al., 1973), a 2 mM increase of stromal Mg^2+^ implies a decrease of total thylakoid Mg^2+^ concentration by 30 mM. This suggests the involvement of a high capacity Mg^2+^-transporting system in the thylakoid membrane, most likely an Mg^2+^-permeable channel (Figure 1).

Patch-clamp study of Pottosin and Schönknecht (1996) revealed the presence of a non-selective cation channel in 75% of patches, derived from intact spinach thylakoids. This (here: ICTCC) intermediate conductance (55 pS in symmetric 100 mM KCl) thylakoid channel was virtually impermeable for Cl^−^, and has a lower conductance, but comparable relative permeability for Ca^2+^ and Mg^2+^ as compared to K^+^. ICTCC appears to be relatively robust, as similar channel activity could be registered also upon reconstitution of spinach thylakoids in giant liposomes or artificial bilayers (Enz et al., 1993; Li et al., 1996). A similar channel was also reported in a direct patch-clamp study on swollen thylakoids from pea leaves (Hinnah and Wagner, 1998). ICTCC was weakly voltage-dependent, with an increase of open probability from 0.1 at −50 to ~0.5 at +50 mV (stroma *minus* lumen). Thus, under light conditions (stromal side made more negative, voltage gradually declining to a steady state), time-averaged current through a single ICTCC would be approximately constant (~−0.3 pA), as a decrease of electrical driving force would be compensated by the increase of open probability (Pottosin and Schönknecht, 1996). As a permeability of ICTCC for K^+^ and Mg^2+^ is comparable, ICTCC-mediated K^+^ and Mg^2+^ fluxes ratio will be determined by the ratio between luminal K^+^ and Mg^2+^ activities. Assuming that it is 10:1, which is probably an upper estimate for the Mg^2+^ flux fraction, a single ICTCC will increase total stromal Mg^2+^ by 1 mM in just 20 s. Free stromal Mg^2+^ increased by 1 mM in the light in 5–10 min (Ishijima et al., 2003). However, only 5% of the chloroplast Mg^2+^ is free, whereas 95% is bound to the thylakoid surface, phosphorylated compounds and dicarboxylates (Portis, 1981). Therefore, the ICTCC—mediated Mg^2+^ flux may account for observed light-induced increase in stromal free Mg^2+^. A curious feature of the light-induced Mg^2+^ influx to the stroma is its insensitivity to La^3+^ and a high sensitivity to ruthenium red (Ishijima et al., 2003), which should be addressed by a direct test with ICTCC.

A light-induced increase of free stromal Mg^2+^, although not being large by absolute value, is essential for activation of the two key enzymes of CO_2_ fixation cycle, fructose -1,6-bisphosphatase (FBPase) and sedoheptulose-1,7- bisphosphatase, SBPase (Portis et al., 1977; Hertig and Wolosiuk, 1980; Wolosiuk et al., 1982). Mg^2+^-deficient plants are characterized by extreme light sensitivity and chlorotic lesions. This was associated with the impairment of the CO_2_ fixation cycle, limiting the overall photosynthetic rate, over-reduction of the electron transport chain, and consequent photodamage due to ROS accumulation (Cakmak and Kirkby, 2008). So far, ICTCC represents the only known Mg^2+^-permeable channel in thylakoids. Important regulatory role of light-induced Mg^2+^ increase on stromal metabolism calls for further studies on the ICTCC molecular identity, distribution, pharmacology, and functional contribution (e.g., existence of alternative ways of Mg^2+^ shuttling between cytosol and stroma).

A separate, potentially more important perspective for ICTCC, is related to its Ca^2+^-permeability. In the last years, ample evidence for the accompaniment of responses to different stresses of the cytosolic Ca^2+^ signal by stromal Ca^2+^ increase has been obtained (Nomura and Shiina, 2014). Moreover, in several occasions stromal Ca^2+^ occurred simultaneously to cytosolic Ca^2+^ increase or even preceded it (Nomura et al., 2012). Stromal Ca^2+^ may be increased by a voltage-driven uniport across the envelope from the cytosol (Roh et al., 1998). As at the rest cytosolic and stromal Ca^2+^ levels are approximately the same, ~150 nM (Johnson et al., 1995), Ca^2+^ uptake into stroma requires a negative potential difference across the envelope, which is the case, especially under light (see below). However, this route may have its limitations. For instance, although light-induced Ca^2+^ uptake to the stroma was readily recorded (Muto et al., 1982), this did not lead to a measurable stromal free Ca^2+^ increase, due to increased stromal Ca^2+^ buffering capacity at more alkaline pH on the light and/or efficient Ca^2+^ uptake by energized thylakoids, which occurs via Ca^2+^/H^+^ antiport mechanism (Ettinger et al., 1999). So, alternatively, passive Ca^2+^ release from thylakoid lumen to stroma may be considered as a cause of a stromal Ca^2+^ signal. A possible role of ICTCC in it requires further elucidation.

## Other channels

Light-induced K^+^ influx into the stroma, together with anion efflux via anion-selective channels of the thylakoid membrane, is indisputably important for electric balance of the light-driven H^+^ uptake by thylakoids, especially at physiologically relevant K^+^ concentrations (Hind et al., 1974). Activity of thylakoid anion or cation channels, due to the opposite direction of respective ion fluxes, will cause thylakoid swelling or shrinkage under light. Thus, volume control may be one of the reasons for the implementation of approximately equivalent fluxes of cations and anions across the thylakoid membrane (Figure 1). The activity of ICTCC may be relevant for a mediation of the light-induced K^+^ flux, but perhaps insufficient, as the whole thylakoid cation current has to be in the range of several pA. Recently, a tandem-pore K^+^-selective channel (TPK3) was localized in *Arabidopsis* stromal thylakoid lamellae (Carraretto et al., 2013). Silencing of TPK3 resulted in *Arabidopsis* plants with reduced growth and altered thylakoid morphology. More specifically, TPK3-silenced plants displayed a reduced non-photochemical quenching, due to a decrease of a steady state ΔpH between stroma and lumen (hence, more alkaline luminal pH). Thus, TPK3 has a strong impact on the steady state p.m.f. partitioning at the light. Preliminary characterization revealed that recombinant TPK3 forms a voltage-independent Ba^2+^-sensitive and TEA^+^ insensitive channel, with a conductance of ~30 pS. It conducts K^+^, but seems to be not perfectly selective for K^+^ over Cl^−^ and its relative selectivity for physiologically relevant cations is unknown. TPK3 likely requires Ca^2+^ for its activity, but whether it is regulated by Ca^2+^ within its physiological range of free concentrations remains to be elucidated. It is instructing that two other explored members of TPK family in *Arabidopsis*, TPK1, and TPK4, are activated by cytosolic H^+^ (Allen et al., 1998; Becker et al., 2004). Preliminary data by Carraretto et al. (2013) demonstrated a strong stimulation of the reconstituted TPK3 by the acidification from pH 7.4 to 6.75, but the sidedness of the effect is unclear. Bearing the large changes of pH in stroma and lumen upon light-dark transitions in mind, the pH-dependence of TPK3 should be addressed. Last, but not least: it is highly desirable to demonstrate the TPK3 activity *in situ*, i.e., in thylakoid blebs of *Arabidopsis*.

For the sake of completeness, an electroneutral (hence, not detectable by the patch-clamp) K^+^/H^+^ antiporter KEA3, localized in the thylakoid membrane, should be considered (Figure 1). It works oppositely to TPK3, reducing the ΔpH gradient across the thylakoid membrane and takes K^+^ up into the lumen (Kunz et al., 2014). Its activity is very essential upon naturally occurring intermittent light conditions. Specifically, upon the transition from a high to a low light condition, KEA3 accelerates the ΔpH dissipation and luminal pH increase, which led to the recovery of the photosystem II quantum efficiency (Armbuster et al., 2014). The presence of two K^+^ transporting systems, TPK3, and KEA3, mediating K^+^ transport in opposite directions and oppositely affecting the lumen acidification is analogous to the presence of a K^+^-permeable channel and KEA1/2 in the inner envelope (see below); a balance between their opposite activities is essential for the chloroplast volume regulation and pH homeostasis (Kunz et al., 2014).

## Envelope ion and solute channels

### Outer envelope contains large-conductance porins

Outer envelope (OE) is an important interface between cytosol and chloroplast. The classical view on OE is that it is a low selective molecular sieve, which is freely permeable to small molecules and proteins with molecular weights of up to 10 kDa (Flügge and Benz, 1984). This view could be reconsidered, however, in light of finding of relatively high cation-selective (P_K+_/P_Cl−_ = 14) porin-like channels OEP23 and OEP37 (Goetze et al., 2006, 2015), and OEP16, a large conductance channel with a striking substrate selectivity for amines and amine acids (Pohlmeyer et al., 1997; Steinkamp et al., 2000; Duy et al., 2007; Pudelski et al., 2010). Moreover, OEP16 excludes sugars, which, basing on their size should pass the channel pore if it was a simple sieve (Pohlmeyer et al., 1997). It was demonstrated for at least one OEP16 isoform, OEP16.2, preferentially expressed in seeds, that its lack of function caused a metabolic imbalance (primarily, changes in free amino acid contents), ABA-hypersensitivity, and early germination (Pudelski et al., 2012). It is also notable, that a relatively weakly selective OEP21 could change its rectification and ionic selectivity from weakly anionic to cationic one upon binding of ATP and phosphorylated carbohydrates exclusively from the intermembrane space (Bölter et al., 1999). This fact argues for a possibility that OE may exert barrier functions for a metabolite exchange between chloroplast and the rest of the cell (Flügge, 2000; Soll et al., 2000; Breuers et al., 2011). As it follows then, the intermembrane space between OE and inner envelope (IE) may represent a compartment on its own, with properties different from those of cytosol. Finally, OEP24 represents the only porin with a low ion selectivity and a broad range of transporting substrates, including amino acids, sugars, and phosphorylated sugars, dicarboxylates, ATP, and inorganic cations (Bölter et al., 1999). It appears that OEP24 relative expression is correlated with a requirement of a higher metabolic flux across the envelope (Bräutigam et al., 2008; Breuers et al., 2011), at which condition its function could not be compensated by higher selective porins. Interestingly, upon heterologous expression in yeast, OEP24 is targeted to the outer envelope membrane of mitochondria, where it can compensate the lack of function of the VDAC (Röhl et al., 1999), principle mitochondrial porin, which controls ions and metabolite traffic across the outer mitochondrial membrane (Colombini, 2012a). Most of the OE porins display bell-shaped voltage dependence, being open at voltages close to zero and closing at large potentials of either sign (Pohlmeyer et al., 1997, 1998; Bölter et al., 1999; Goetze et al., 2006). It is believed that this property does not have a physiological importance, as there is no potential drop across the OE except for a (supposedly small) Donnan potential difference due to accumulation of large weakly permeable anionic species in the intermembrane space. There are some studies for mitochondria, however, which estimate the Donnan potential difference could be as large as 20–40 mV (cytosol positive; Porcelli et al., 2005). At +25 mV VDAC is switched to a subconductance state, and VDAC-mediated ATP transport stops (Colombini, 2012b). A similar situation may exist with OEP21, which is blocked by ATP and at the same time seems to transport it in a “tight fit manner” (Hemmler et al., 2006). Notably, OEP21 has the steepest voltage dependence among chloroplast porins, which is similar to that of VDAC, and at large potentials it is switched to a subconductance state of about 20% of maximal conductance (Bölter et al., 1999). A maximum of time-averaged conductance for OEP21 is observed at +25 mV, whereas at zero voltage it is close to minimal one. Therefore, for this porin, in contrast to other OEPs, it would be essential, whether the voltage difference across the OE is zero or +25 mV (the value, close to that for the Donnan potential difference in mitochondria).

Proteomic analysis of the OE demonstrated that OE is enriched in five non-redundant transport proteins: an ABC transporter with unknown function and aforementioned OEP 16, 21, 24, and 37, plus a protein translocon channel, formed by TOC75 (Gutierrez-Carbonell et al., 2014). Although this list may be non-exhaustive, it can serve as a first-aid guide for the identification of porins, measured in intact chloroplast membranes. Large conductance weakly selective (P_K_+/P_Cl−_ ~ 2.3) porins, with a bell-shaped voltage dependence could be detected in intact chloroplasts of higher plants (Muñiz et al., 1995). All these properties are reminiscent of those for pea OEP24 (Pohlmeyer et al., 1998). Measurements on intact chloroplasts of a Charophyte alga *Nitellopsis obtusa* revealed two types of large-conductance porin-like channels and an anion selective channel (Pottosin, 1992). Anion selective-channel by its conductance and voltage dependence was very similar to its counterpart in the thylakoid membrane (Pottosin and Schönknecht, 1995a), albeit being less selective for Cl^−^ over K^+^, P_Cl−_/${\text{P}}_{K}^{+}$ ~ 12. A weakly selective anion channel with a similar conductance and identical voltage dependence was registered upon reconstitution of spinach envelope membranes into lipid bilayers (Viérick et al., 2003). These findings corroborate the report on the localization of ClC-f in the OE (Teardo et al., 2005), although an expected “double-barreled” gating of this tentative channel remains to be demonstrated functionally. The most abundant weakly selective cation porin in a *Nitellopsis* chloroplast was reminiscent of VDAC by its voltage dependence, substates occurrence, and effect exerted by the synthetic König's polyanion, known gating modulator of VDAC (Colombini et al., 1987; Pottosin, 1993). When compared to pea porins, its voltage-dependent behavior (position of maximum at ~+20 mV, steepness, closure to a subconductance state of about 20% of maximal at large potentials of either sign) better fits that of OEP21. OEP21 displayed cation selectivity only in the presence of submillimolar to low millimolar ATP in the intermembrane space (Bölter et al., 1999). Such a condition, however, might also naturally occur in experiments on intact chloroplasts on *Nitellopsis*. Summarizing, patch-clamp measurements on relatively large (about 10 μm) and robust chloroplasts of *N. obtusa* revealed ion channels, which, on the basis of their activity and large conductance, are more likely to be located in the OE. However, working on smaller chloroplasts from higher plants, we were forced to make different assumptions on the patch configuration upon stable recording.

### Is it possible to measure ion channel activity from the inner envelope of an intact chloroplast?

**Figure 2:**
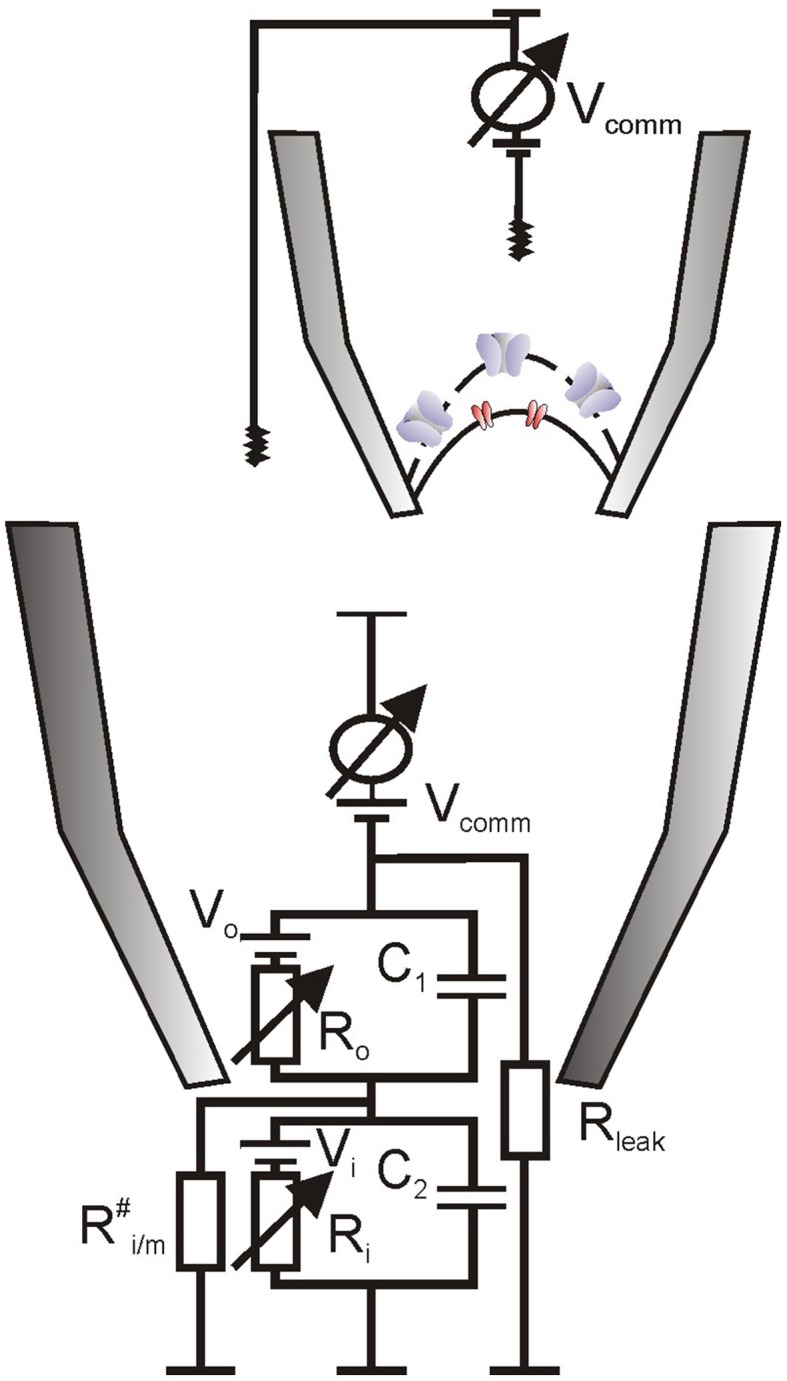
**Hypothetical equivalent electrical circuit for recording on a sandwich-like envelope patch**. It is assumed that the intermembrane space was tightly sealed (${\text{R}}_{i/m}^{\#}\text{ >>}$ R_o_) and that inner envelope electrical resistance was much higher than of the outer envelope, R_i_ >> R_o_ (due to the presence of multiple porins in the latter), so that command voltage (V_comm_) dropped almost entirely across the inner envelope membrane patch (V_comm_ ~V_i_). R_o_ represents the access resistance, which was ~1 GOhm for measurements on pea chloroplasts, equivalent to a presence of few (2–3) open porins in the outer patch membrane. Average patch capacitance was about 0.35 pF; on the basis of specific capacitance for biological membranes (~1 μF/cm^2^) this transforms to 35 μm^2^ or 70% of the pea chloroplast surface, additional argument for the presence of a double membrane vesicle in the patch (Pottosin et al., 2005).

Patch-clamp measurements of porin-like channels in attached configuration on intact pea chloroplasts could be done only within a short period, due to a spontaneous patch isolation and chloroplast lysis (Pottosin et al., 2005). Before the collapse, currents through porin-like channels were distorted and decreased in amplitude as if a membrane vesicle was formed on the tip of the pipette (Hamill et al., 1981). We believed that, due to a very close alignment of the OE and IE membranes (5 nm space) and existence of tight contact sites between the two envelope membranes, it was hardly possible that this vesicle was formed by the OE alone, stripped from the IE. Most likely, the sealing of the intermembrane space took place upon patch isolation, so that a sandwich-like arrangement of OE and IE resulted (Figure 2). Insulation of the transmembrane space is required for a switch of a recording on the OE to that on the IE. As electrical resistance of the OE, due to the presence of high conductance pores, is much lower than that of the IE, command voltage will fall mainly across the IE and the IE will limit the overall ion flux. The fraction of voltage drop across the OE would be close to zero, so porins would be mainly locked in the open state, fixing this condition over the whole experiment in inside-out patch configuration. The same conclusion was drawn by van den Wijngaard and Vredenberg (1997; van den Wijngaard et al., 2000)—another group, which succeeded in stable patch-clamp recordings on the intact chloroplast envelope of pea.

### Fast-activated cation channel in the pea envelope membrane

Spontaneous isolation of the envelope patch resulted in a quite different pattern of channels' activity. The dominant smaller conductance channels were rapidly activated (τ~ 1–2 ms) by large membrane polarizations of either sign and were highly selective for K^+^ over Cl^−^, P_Cl−_/${\text{P}}_{K}^{+}$ < 0.03, but weakly differentiated between K^+^, Na^+^, Ca^2+^, or Mg^2+^ (Pottosin et al., 2005). It was termed FACC, for the fast activating chloroplast cation channel (Figure 1). By its permeability to divalent cations and conductance value, FACC may be compared with the glutamate receptor iGLR3 channel, which was found in the IE of *Arabidopsis* chloroplasts and assayed upon fusion of IE vesicles with an artificial planar lipid bilayer (Teardo et al., 2010). It should be interesting, then, to test effects of iGLR3 channel agonists and antagonists against FACC. Physiologically, FACC will mainly mediate the exchange of K^+^ (and Na^+^) across the envelope, because free concentrations of Ca^2+^ and Mg^2+^ at both envelope sides are lower than those for K^+^ by a factor of 10^6^ and 10^2^, respectively. FACC displayed a U-shaped voltage dependence of the open probability, which was reminiscent of this for so-called fast vacuolar channel, FV (Tikhonova et al., 1997). However, current via FV channel was almost completely abolished by sub- mM cytosolic Ca^2+^ or Mg^2+^ (Tikhonova et al., 1997; Brüggemann et al., 1999) and the FACC-mediated currents could be measured in the presence of high concentrations of these cations. Yet, a reduction of cytosolic Ca^2+^ from 2 mM to physiological level of 200 nM caused an increase of the FACC open probability almost 10-fold in the physiological voltage range (Pottosin et al., 2005). Trans-envelope voltage difference is negative at stromal side and decreased by absolute value upon an increase of cytosolic KCl concentration, from −110 mV at 5 mM to −20 mV at −100 mM KCl under light; in dark conditions voltage difference is depolarized by ~20 mV (Demmig and Gimmler, 1983). FACC also sensed stromal pH changes within the range, correspondent to light- dark transition: its open probability increased several-fold upon the pH change from 8 to 7.3 (Pottosin et al., 2005). Such a behavior may be expected from a channel, which tended to oppose stromal acidification.

An alkaline stromal pH (~8) is optimal for the activity of FBPase and SBPase, whereas at pH below 7.3 the turnover rate of the CO_2_ fixation cycle became very low (Werdan et al., 1975; Chardot and Meunier, 1991). Light-driven uptake of H^+^ and a parallel flux of Cl^−^ across the thylakoid membrane were extended to a decrease of activity of these ions in the cytosol, a stable one for Cl^−^ and a transient one for H^+^ (Thaler et al., 1991). The reversibility of the light-induced cytosol alkalinization may argue for the existence of an active mechanism of the H^+^ extrusion from stroma across the envelope. Functional evidence suggested that H^+^ evacuation from stroma is mediated by yet non-identified IE primary H^+^ pump (Maury et al., 1981; Berkowitz and Peters, 1993). Function of an electrogenic H^+^ pump will polarize the trans-envelope potential (Figure 1), which calls for a balancing counterion flux. It is long known that stromal acidification may be reversibly induced by depletion of the external medium of monovalent cations, K^+^ and Na^+^, which could act, therefore, as counterions for active H^+^ extrusion (Demmig and Gimmler, 1983; Wu et al., 1991; Wu and Berkowitz, 1992; Heiber et al., 1995). The respective K^+^ influx to and H^+^ efflux from stroma are in the range of 100 and 20 nmol/min per mg of chlorophyll at 10–20 and 100 mM external K^+^, respectively (Demmig and Gimmler, 1983; Wu et al., 1991); a decreased K^+^ influx at higher external K^+^ may be dealt with by a decrease of the K^+^-driving force (trans-envelope voltage difference). These values transform to electrical current of 0.2 and 0.04 pA per single chloroplast; the latter value (for 100 mM cytosolic KCl) may be more physiologically relevant. At physiological pH, Ca^2+^ and voltage a single FACC mediated ~1 pA current and had an open probability of 1–2% (Pottosin et al., 2005), which implies that a single FACC conducted the time-averaged K^+^ current of approximately 10–20 fA. Thus, the activity of few FACC channels as typically observed in isolated chloroplast patches (Pottosin et al., 2005) might be sufficient to balance the IE pump-mediated H^+^ extrusion. As will be discussed below, the chloroplast volume appears to be finely balanced by channel-mediated K^+^ influx and K^+^/H^+^ antiporters- mediated K^+^ efflux across the envelope (Figure 1). On the quantitative basis, therefore, we believe that high-conductance and poorly regulated K^+^ permeable channels, reported by others to be present in the IE (Mi et al., 1994), are likely artifacts of reconstitution procedures. Indeed, a single channel of this type will conduct a time-averaged current of few pA, which is by two orders of magnitude above the demand. It will collapse the transenvelope voltage difference and will always overweight the K^+^ flux, generated by IE K^+^/H^+^ antiporters, making their contribution to volume control insignificant and the overall function (stromal acidification) counterproductive.

An important note should be made on the thermodynamics of K^+^(Na^+^)/H^+^ exchange across the envelope. H^+^ extrusion from stroma to cytosol is against the H^+^ gradient and may be only active. This active transport will fuel a downhill channel-mediated K^+^(Na^+^) uptake into the stroma. The direction of transports, thus, is exactly opposite to those mediated by conventional K^+^(Na^+^)/H^+^ antiporters. In the chloroplast IE two K^+^/H^+^ antiporters, *KEA1* and *KEA2*, and Na^+^/H^+^ antiporter *NHD1* were identified (Kunz et al., 2014). These cation/H^+^ antiporters transport H^+^ downhill *into* the stroma (hence, acidifying it), and *export* K^+^ (or Na^+^) to the cytosol (thus, assisting the chloroplast regulated volume decrease). Consequently, triple *kea1kea2nhd1* lack-of-function mutants displayed a severe phenotype, with swollen or broken chloroplasts (Kunz et al., 2014). These two different types of the IE K^+^(Na^+^)/H^+^ antiport systems, one based on K^+^(Na^+^)/H^+^ antiporters and another consisting of a non-selective cation channel and of an H^+^ pump, should be both essential for stromal pH control and chloroplast volume regulation, exerting the effects opposite one to another. It was shown that osmotic adjustment in chloroplasts, crucial for the photosynthesis efficiency, requires transport of organic osmolytes in addition to inorganic ions and that the permeability of the IE for sugars is increased upon acute water stress (Robinson, 1985; McCain, 1995). This argues for the activity of mechanosensitive channels, which pores are wide enough to transport sugars. As far as we know, mechanical stretch-stimulation was never tested upon *in situ* patch-clamp measurements on intact chloroplasts, which may be addressed for future studies. However, the presence of MscS-like MSL 2 and 3 channels in the IE and their impact on the chloroplast volume was demonstrated, suggesting that they form functional mechanosensitive channels in *Arabidopsis* (Haswell and Meyerowitz, 2006). Ionic selectivity of MSL 2 and 3 is unknown, whereas a related MSC1 channel in *Chlamydomonas* chloroplasts is selective for anions over K^+^ (Nakayama et al., 2007). On the other hand, FACC was permeable to N-methyl D-glucamine (Pottosin et al., 2005), and, therefore, possesses a pore wide enough to transport monosaccharides.

### Anion channel (PIRAC) in the pea envelope membrane

In an interesting variation with our data, direct patch-clamping of pea chloroplasts by other group revealed that the envelope conductance in their case was dominated by a voltage-independent 50 pS anion channel (van den Wijngaard and Vredenberg, 1997, 1999; van den Wijngaard et al., 1999). Its open probability was negatively regulated by protein precursors (preferredoxin), so it was named PIRAC (for protein import related anion channel) and its interaction with the IE protein translocon (Tic) was considered, although it probably did not form a part of the Tic pore (van den Wijngaard and Vredenberg, 1999; van den Wijngaard et al., 2000). An anion channel has to be present in the IE (and possibly, also in the OE) due to the fact of a large cytosolic Cl^−^ decrease, caused by the light-driven anion uptake by chloroplasts (Thaler et al., 1991). Because PIRAC activity was studied in a relatively narrow voltage range, ±40 mV, where FACC open probability did not exceed 2%, FACC could be easily overlooked. However, how could we, in turn, overlook the activity of PIRAC, which open probability was close to 1? Comparison of our experimental conditions with those employed by van den Wijngaard and co-workers, revealed a single potentially important difference: for some reasons these authors used Mg^2+^-free solutions, whereas in our case minimal free Mg^2+^ concentration at either membrane side was 1 mM (Pottosin et al., 2005). It is well known that the activity of mCS anion channel of the inner mitochondrial membrane is completely abolished by inclusion of 1 mM Mg^2+^ in solutions (Borecký et al., 1997). High intracellular Mg^2+^ also inhibits volume-regulated anion channels (Nilius and Droogmans, 2003). This opens a very interesting perspective, as Mg^2+^ may act as a natural regulator of PIRAC.

## Outlook

*In vitro* studies of individual OE porins, either purified native or recombinant proteins, is undoubtedly a very important source of information on their detailed properties. However, it is believed, albeit not proved experimentally, that all or at least some OEPs are negatively controlled *in vivo* and *in situ*, so that they are closed most of time and open only on demand- the situation, obviously, rather different from their high open probability *in vitro* (Bölter and Soll, 2001; Duy et al., 2007). Direct patch-clamping of the intact chloroplast envelope may be the way to prove or disprove this hypothesis. Up to the moment, only two groups, our lab and that from Prof. Vredenberg were succeeded in direct intact chloroplast patch-clamping, and most of available information for a higher plant chloroplast was obtained on a single species—the pea. The pea played an important role in OEP studies, as OEPs were first identified in this species and only afterwards in *Arabidopsis*. Nevertheless, there is really a need to extend the list of explored species. *Arabidopsis* and rice chloroplasts are on the top of this list. We are unaware of such attempts in the past and may not judge, whether the absence of respective information is due to an extremely low success rate or just unwillingness to try. However, as pea chloroplasts were not uniquely patch-clamp friendly, we believe that it is worth to pursue the patch-clamp trials. We are also thinking that a concurrent implementation of a non-invasive MIFE technique to study ion fluxes on chloroplasts arrays, similar to the previous application of MIFE to studies on microorganisms (Shabala et al., 2006), may be of importance to elucidate the trans-envelope ion transport *in situ*. Our observations on electrical behavior of pea chloroplasts upon patching raised the possibility to register currents from the two envelope membranes in series. This model, of course, requires further proofs. However, a tightly regulated membrane conductance, revealed in our experiments as compared to a vast variety of poorly regulated conductance states in reconstituted studies (Mi et al., 1994; Heiber et al., 1995), appears to be more consistent with barrier properties of the IE. Thylakoid membrane patching, and, in particular, patching native thylakoid membrane of *Arabidopsis*, represents an interesting and potentially rewarding task for the future. Again, available data are too scarce for generalizations. Nevertheless, the dominant anion channel seems to be conserved as it was detected in only two distantly related dicotyledonous species and in alga chloroplasts. Its identity with ClC (ClC-e?), regulation by protons, and pharmacological profile remain to be elucidated. Divalent cation-permeable channel so far was found only in spinach thylakoid membrane (Pottosin and Schönknecht, 1996). Do similar channels exist in thylakoid membranes of other higher plant species? If so, what is their molecular identity and actual roles in stromal Ca^2+^ signaling and control of CO_2_ fixation by Mg^2+^? Finally, an interesting approach of thylakoid transport studies may be the achievement of a whole thylakoid configuration, using a perforated patch technique. It will allow not only to record currents mediated by all present ion channels, but in conjunction with it also measure light-induced electron and H^+^ transport under strictly controlled voltage and pH conditions. This may represent a more direct way to study the effects of pH and voltage on the photosynthetic transfer and to reveal particular contributions of different ion channels into a dynamic control of the thylakoid ΔΨ.

## Author contributions

IP was in charge of general planning, writing, editing, and figures design. OD performed database search, writing, editing, and proof-reading.

### Conflict of interest statement

The authors declare that the research was conducted in the absence of any commercial or financial relationships that could be construed as a potential conflict of interest.

## References

[B1] Allen G. A. Amtmann A. Sanders D. (1998). Calcium-dependent and calcium-independent K^+^ mobilization channels in *Vicia faba* guard cell vacuoles. J. Exp. Bot. 49, 305–318. 10.1093/jxb/49.Special_Issue.305

[B2] Allen J. F. Holmes N. G. (1986). Electron transport and redox titration, in Photosynthesis-Energy Transduction: A Practical Approach, eds Hipkins M. F. Baker N. R. (Oxford: IRL Press), 103–141.

[B3] Armbuster U. Carrillo L. R. Venema K. Pavlovic L. Schmidtmann E. Kornfeld A. (2014). Ion antiport accelerates photosynthetic acclimation in fluctuating light environments. Nat. Comm. 5, 5439 10.1038/ncomms6439 PMC4243252 25451040

[B4] Becker D. Geiger D. Dunkel M. Roller A. Bertl A. Latz A.. (2004). AtTPK4, an *Arabidopsis* tandem-pore K_ channel, poised to control the pollen membrane voltage in a pH- and Ca^2+^-dependent manner. Proc. Natl. Acad. Sci. U.S.A. 101, 15621–15626. 10.1073/pnas.0401502101 15505206 PMC524823

[B5] Berkowitz G. A. Peters J. S. (1993). Chloroplast inner-envelope ATPase acts as a primary H^+^ pump. Plant Physiol. 102, 261–267. 10.1104/pp.102.1.261 12231817 PMC158771

[B6] Block M. A. Douce R. Joyard J. Rolland N. (2007). Chloroplast envelope membranes: a dynamic interface between plastids and the cytosol. Photosyn. Res. 92, 225–244. 10.1007/s11120-007-9195-8 17558548 PMC2394710

[B7] Bölter B. Soll J. (2001). Ion channels in the outer membranes of chloroplasts and mitochondria: open doors or regulated gates? EMBO J. 20, 935–940. 10.1093/emboj/20.5.935 11230117 PMC145478

[B8] Bölter B. Soll J. Hill K. Hemmler R. Wagner R. (1999). A rectifying ATP-regulated solute channel in the chloroplastic outer envelope from pea. EMBO J. 18, 5505–5516. 10.1093/emboj/18.20.5505 10523295 PMC1171619

[B9] Borecký J. Jezek P. Siemen D. (1997). 108-pS channel in brown fat mitochondria might be identical to the inner membrane anion channel. J. Biol. Chem. 272, 19282–19289. 9235923

[B10] Bräutigam A. Hofmann-Benning S. Weber A. P. M. (2008). Comparative proteomics of chloroplast envelopes from C3 and C4 plants reveals specific adaptations of the plastid envelope to C4 photosynthesis and candidate proteins required for maintaining C4 metabolite fluxes. Plant Physiol. 148, 568–579. 10.1104/pp.108.121012 18599648 PMC2528119

[B11] Breuers F. H. H. Bräutigam A. Weber A. P. M. (2011). The plastid outer envelope- a highly dynamic interface between plastid and cytoplasm. Front. Plant Sci. 2:97. 10.3389/fpls.2011.00097 22629266 PMC3355566

[B12] Brüggemann L. I. Pottosin I. I. Schönknecht G. (1999). Cytoplasmic magnesium regulates the fast activating vacuolar cation channel. J. Exp. Bot. 50, 1547–1552. 10.1093/jxb/50.339.1547

[B13] Bulychev A. A. Andrianov V. K. Kurella G. A. Litvin F. F. (1972). Microelectrode measurements of the ransmembrane potential of chloroplasts and its photoinduced changes. Nature 236, 175–177. 10.1038/236175a0

[B14] Bulychev A. A. Antonov V. F. Schevchenko E. V. (1992). Patch-clamp studies of light-induced currents across the thylakoid membrane of isolated chloroplasts. Biochim. Biophys. Acta Bioenerg. 1099, 16–24. 10.1016/0005-2728(92)90182-2

[B15] Cakmak I. Kirkby E. A. (2008). Role of magnesium in carbon partitioning and alleviating photooxidative damage. Physiol. Plant. 133, 692–704. 10.1111/j.1399-3054.2007.01042.x 18724409

[B16] Campo M. L. Tedeschi H. (1985). Protonmotive force and photophosphorylation in single swollen thylakoid vesicles. Eur. J. Biochem. 149, 511–516. 10.1111/j.1432-1033.1985.tb08954.x 2988949

[B17] Carraretto L. Formentin E. Teardo E. Checchetto V. Tomizioli M. Morosinotto T.. (2013). A thylakoid-located two-pore K^+^ channel controls photosynthetic light utilization in plants. Science 342, 114–118. 10.1126/science.1242113 24009357

[B18] Chardot T. Meunier J. -C. (1991). Properties of oxidized and reduced spinach (*Spinacia oleracea*) chloroplast fructose-1,6-bisphosphatase activated by various agents. Biochem. J. 278, 787–781. 10.1042/bj2780787 1654892 PMC1151415

[B19] Checchetto V. Teardo E. Carraretto L. Formentin E. Bergantino E. Giacometti G. M.. (2013). Regulation of photosynthesis by ion channels in cyanobacteria and higher plants. Biophys. Chem. 182, 51–57. 10.1016/j.bpc.2013.06.006 23891570

[B20] Colombini M. (2012a). Mitochondrial outer membrane channels. Chem. Rev. 112, 6373–6387. 10.1021/cr3002033 22979903

[B21] Colombini M. (2012b). VDAC structure, selectivity, and dynamics. Biochim. Biophys. Acta 1818, 1457–1465. 10.1016/j.bbamem.2011.12.026 22240010 PMC3327780

[B22] Colombini M. Yeung C. L. Tung J. König T. (1987). The mitochondrial outer membrane channel, VDAC, is regulated by a synthetic polyanion. Biochim. Biophys. Acta 905, 279–286. 2446659 10.1016/0005-2736(87)90456-1

[B23] Cruz J. A. Sacksteder C. Kanazawa A. Kramer D. M. (2001). Contribution of electric field ΔΨ to steady-state transthylakoid proton motive force *in vitro* and *in vivo*. Control of pmf parsing into ΔΨ and ΔpH by counterion fluxes. Biochemistry 40, 1226–1237. 10.1021/bi0018741 11170448

[B24] Demmig B. Gimmler H. (1983). Properties of the isolated intact chloroplast at cytoplasmic K^+^ concentrations.I. Light-induced cation uptake into intact chloroplasts is driven by an electrical potential difference. Plant Physiol. 73, 169–174. 10.1104/pp.73.1.169 16663169 PMC1066428

[B25] Duy D. Soll J. Philippar K. (2007). Solute channels of the outer membrane: from bacteria to chloroplasts. Biol. Chem. 388, 879–889. 10.1515/BC.2007.120 17696771

[B26] Enz C. Steinkamp T. Wagner R. (1993). Ion channels in the thylakoid membrane (A Patch Clamp Study). Biochim. Biophys. Acta 1143, 67–76. 10.1016/0005-2728(93)90217-4

[B27] Ettinger W. F. Clear A. M. Fanning K. J. Peck M. L. (1999). Identification of a Ca^2+^/H^+^ antiport in the plant chloroplast thylakoid membrane. Plant Physiol. 119, 1379–1385. 10.1104/pp.119.4.1379 10198097 PMC32023

[B28] Fischer S. Gräber P. (1999). Comparison of ΔpH- and ΔΨ-driven ATP synthesis catalyzed by the H^+^-ATPases from *Escherichia coli* or chloroplasts reconstituted into liposomes. FEBS Lett. 457, 327–332. 10471802 10.1016/s0014-5793(99)01060-1

[B29] Flügge U. Benz R. (1984). Pore forming activity in the outer membrane of the chloroplast envelope. FEBS Lett. 169, 85–89. 10.1016/0014-5793(84)80294-X

[B30] Flügge U. I. (2000). Transport in and out of plastids: does the outer envelope membrane control the flow? Trends Plant Sci. 5, 135–137. 10.1016/S1360-1385(00)01578-8 10740292

[B31] Gimmler H. Schäfer G. Heber U. (1974). Low permeability of the chloroplast envelope towards cations, in Proceedings of the 3rd International Congress on Photosynthesis Research, Vol. 3, ed Avron M. (Amsterdam: Elsevier), 1381–1392.

[B32] Goetze T. A. Patil M. Jeshen I. Bölter B. Grahl S. Soll J. (2015). Oep23 forms an ion channel in the chloroplast outer enveliope. BMC Plant Biol. 15:47. 10.1186/s12870-015-0445-1 25849634 PMC4331141

[B33] Goetze T. A. Philippar K. Ilkavets I. Soll J. Wagner R. (2006). OEP37 is a new member of the chloroplast outer membrane ion channels. J. Biol. Chem. 281, 17989–17998. 10.1074/jbc.M600700200 16624824

[B34] Gould S. B. Waller R. F. McFadden G. I. (2008). Plastid evolution. Annu. Rev. Plant Biol. 59, 491–517. 10.1146/annurev.arplant.59.032607.092915 18315522

[B35] Gutierrez-Carbonell E. Takahashi D. Lattanzio G. Rodríguez-Celma J. Kehr J. Soll J.. (2014). The distinct functional roles of the inner and outer chloroplast envelope of pea (*Pisum sativum*) as revealed by proteomic approaches. J. Proteome Res. 13, 2941–2953. 10.1021/pr500106s 24792535

[B36] Hamill O. P. Marty A. Neher E. Sakmann B. Sigworth F. J. (1981). Improved patch clamp technique for high-resolution current recording from cells and cell free patches. Pfluegers Arch. 391, 85–100. 10.1007/BF00656997 6270629

[B37] Haswell E. S. Meyerowitz E. M. (2006). MscS-like proteins control plastid size and shape in Arabidopsis thaliana. Curr. Biol. 16, 1–11. 10.1016/j.cub.2005.11.044 16401419

[B38] Heiber T. Steinkamp T. Hinnah S. Schwarz M. Flügge U. I. Weber A.. (1995). Ion channels in the chloroplast envelope membrane. Biochemistry 34, 15906–15917. 10.1021/bi00049a005 8519747

[B39] Heldt H. W. Werdan K. Milovancev M. Geller G. (1973). Alkalinization of the chloroplast stroma caused by light-dependent proton flux into the thylakoid lumen. Biochim. Biophys. Acta 314, 224–241. 10.1016/0005-2728(73)90137-0 4747067

[B40] Hemmler R. Becker T. Schleiff E. Bölter B. Stahl T. Soll J.. (2006). Molecular properties of Oep21, an ATP-regulated anion- selective solute channel from the outer chloroplast membrane. J. Biol. Chem. 281, 12020–12029. 10.1074/jbc.M513586200 16473880

[B41] Herick K. Krämer R. Lúhring H. (1997). Patch clamp investigation into the phosphate carrier from *Saccharomyces cerevisiae* mitochondria. Biochim. Biophys. Acta 1321, 207–220. 10.1016/S0005-2728(97)00050-9 9393638

[B42] Hertig C. Wolosiuk R. A. (1980). A dual effect of Ca^2+^ on chloroplast fructose-1,6-bisphosphatase. Biochem. Biophys. Res. Commun. 97, 325–333. 10.1016/S0006-291X(80)80171-9 6257242

[B43] Hind G. Nakatani H. Y. Izawa S. (1974). Light-dependent redistribution of ions in suspensions of chloroplast thylakoid membranes. Proc. Natl. Acad. Sci. U.S.A. 71, 1484–1488. 10.1073/pnas.71.4.1484 4524652 PMC388254

[B44] Hinnah S. Wagner R. (1998). Thylakoid membranes contain a high-conductance channel. Eur. Biochem. J. 253, 606–613. 10.1046/j.1432-1327.1998.2530606.x 9654056

[B45] Inoue K. (2007). The chloroplast outer envelope membrane: the edge of light and excitement. J. Integrat. Plant Biol. 49, 1100–1111. 10.1111/j.1672-9072.2007.00543.x

[B46] Ishijima S. Uchibori A. Takagi H. Maki R. Ohnishi M. (2003). Light-induced increase in free Mg^2+^ concentration in spinach chloroplasts: measurement of free Mg^2+^ by using a fluorescent probe and necessity of stromal alkalinization. Arch. Biochem. Biophys. 412, 126–132. 10.1016/S0003-9861(03)00038-9 12646275

[B47] Johnson C. H. Knight M. R. Kondo T. Masson P. Sedbrook J. Haley A.. (1995). Circadian oscillations of cytosolic and chloroplastic free calcium in plants. Science 269, 1863–1865. 10.1126/science.7569925 7569925

[B48] Kirchhoff H. (2008). Molecular crowding and order in photosynthetic membranes. Trends Plant Sci. 13, 201–207. 10.1016/j.tplants.2008.03.001 18407783

[B49] Kirchhoff H. Mukherjee U. Galla H. -J. (2002). Molecular architecture of the thylakoid membrane: lipid diffusion space for plastoquinone. Biochemistry 41, 4872–4882. 10.1021/bi011650y 11939782

[B50] Klughammer C. Siebke K. Schreiber U. (2013). Continuous ECS-indicated recording of the proton-motive charge flux in leaves. Photosynth. Res. 117, 471–487. 10.1007/s11120-013-9884-4 23860827 PMC3825596

[B51] Kramer D. M. Avenson T. J. Edwards G. E. (2004). Dynamic flexibility in the light reactions of photosynthesis governed by both electron and proton transfer reactions. Trends Plant Sci. 9, 349–357. 10.1016/j.tplants.2004.05.001 15231280

[B52] Kramer D. M. Cruz J. A. Kanazawa A. (2003). Balancing the central roles of the thylakoid proton gradient. Trends Plant Sci. 8, 27–32. 10.1016/S1360-1385(02)00010-9 12523997

[B53] Kunz H. H. Gierth M. Herdean A. Satoh-Cruz M. Kramer D. M. Spetea C.. (2014). Plastidial transporters KEA1, -2, and -3 are essential for chloroplast osmoregulation, integrity, and pH regulation in *Arabidopsis*. Proc. Natl. Acad. Sci. U.S.A. 111, 7480–7485. 10.1073/pnas.1323899111 24794527 PMC4034250

[B54] Labarca P. Latorre R. (1992). Insertion of ion channels into planar lipid bilayers by vesicle fusion, in Methods in Enzymology: Vol. 207, Ion Channels, eds Rudy B. Iverson L. E. (New York, NY: Academic Press), 447–463. 10.1016/0076-6879(92)07032-j 1382196

[B55] Li L. Yan J. Tang Z. (1996). Direct measurement of the K^+^-channel and its ion selectivity in the thylakoid membrane from spinach (*Spinacea oleracea* L.) leaves. Acta Bot. Sin. 38, 692–698.

[B56] Marmagne A. Vinauger-Doyard M. Monachello D. Falcon de Longevialle A. Charon C. Allot M.. (2007). Two members of the *Arabidopsis* CLC (chloride channel) family, AtCLCe and AtCLCf, are associated with thylakoid and Golgi membranes, respectively. J. Exp. Bot. 58, 3385–3393. 10.1093/jxb/erm187 17872921

[B57] Maury W. J. Huber S. C. Moreland D. E. (1981). Effects of magnesium on intact chloroplasts. II. Cation specificity and involvement of the envelope ATPase in (sodium) potassium/proton exchange across the envelope. Plant Physiol. 68, 1257–1263. 10.1104/pp.68.6.1257 16662089 PMC426084

[B58] McCain D. C. (1995). Combined effects of light and water stress on chloroplast volume regulation. Biophys. J. 69, 1105–1110. 10.1016/S0006-3495(95)79984-2 8519964 PMC1236338

[B59] Mi F. Berkowitz G. A. Peters J. S. (1994). Characterization of a chloroplast inner envelope K^+^ channel. Plant Physiol. 105, 955–964. 10.1104/pp.105.3.955 8058841 PMC160746

[B60] Miller C. (2006). ClC chloride channels viewed through a transport lens. Nature 220, 484–489. 10.1038/nature04713 16554809

[B61] Mitchell P. (1966). Chemiosmotic coupling in oxidative and photosynthetic phosphorylation. Biol. Rev. Cambridge Phil. Soc. 41, 445–502. 10.1111/j.1469-185X.1966.tb01501.x 5329743

[B62] Monachello D. Allot M. Oliva S. Krapp A. Daniel-Videle F. Barbier-Brygoo H.. (2009). Two anion transporters AtClCa and AtClCe fulfill interconnecting but not redundant roles in nitrate assimilation pathways. New Phytol. 183, 88–94. 10.1111/j.1469-8137.2009.02837.x 19402883

[B63] Muñiz J. M. Pottosin I. I. Sandoval L. (1995). Patch-clamp study of vascular plant chloroplasts: ion channels and photocurrents. J. Bioenerg. Biomembr. 27, 249–258. 10.1007/BF02110040 7592572

[B64] Muto S. Izawa S. Miyachi S. (1982). Light-induced Ca^2+^ uptake by intact chloroplasts. FEBS Lett. 139, 250–254. 10.1016/0014-5793(82)80863-6

[B65] Nakayama Y. Fujiu K. Sokabe M. Yoshimura K. (2007). Molecular and electrophysiological characterization of a mechanosensitive channel expressed in the chloroplasts of *Chlamydomonas.* Proc. Natl. Acad. Sci. U.S.A. 104, 5883–5888. 10.1073/pnas.0609996104 17389370 PMC1851586

[B66] Nilius B. Droogmans G. (2003). Amazing chloride channels: an overview. Acta Physiol. Scand. 177, 119–147. 10.1046/j.1365-201X.2003.01060.x 12558550

[B67] Nomura H. Komori T. Uemura S. Kanda Y. Shimotani K. Nakai K.. (2012). Chloroplast-mediated activation of plant immune signalling in *Arabidopsis*. Nat. Commun. 3, 926. 10.1038/ncomms1926 22735454

[B68] Nomura H. Shiina T. (2014). Calcium signaling in plant endosybiotic organelles: mechanism and role in physiology. Mol. Plant 7, 1094–1104. 10.1093/mp/ssu020 24574521

[B69] Pelzer D. J. McDonald T. F. Pelzer S. (1993). Reconstitution of muscle calcium channel function in bilayer membranes: from the first steps to results, in Methods in Pharmacology: Vol. 7, Molecular and Cellular Biology of Pharmacological Targets, eds Glossmann H. Striessnig J. (New York, NY: Springer Sci.), 99–140.

[B70] Pfeil B. E. Schoefs B. Spetea C. (2014). Function and evolution of channels and transporters in photosynthetic membranes. Cell. Mol. Life Sci. 71, 979–998. 10.1007/s00018-013-1412-3 23835835 PMC3928508

[B71] Pohlmeyer K. Soll J. Grimm R. Hill K. Wagner R. (1998). A high-conductance solute channel in the chloroplastic outer envelope from pea. Plant Cell 10, 1207–1216. 10.1105/tpc.10.7.1207 9668138 PMC144050

[B72] Pohlmeyer K. Soll J. Steinkamp T. Hinnah S. Wagner R. (1997). Isolation and characterization of an amino acid-selective channel protein present in the chloroplastic outer envelope membrane. Proc. Natl. Acad. Sci. U.S.A. 94, 9504–9509. 10.1073/pnas.94.17.9504 9256512 PMC23240

[B73] Porcelli A. M. Ghelli A. Zanna C. Pinto P. Rizzuto R. Rugolo M. (2005). pH difference across the outer mitochondrial membrane measured with a green fluorescent protein mutant. Biochem. Biophys. Res. Commun. 326, 799–804. 10.1016/j.bbrc.2004.11.105 15607740

[B74] Portis A. Heldt H. W. (1976). Light dependent changes of the Mg^2+^ concentration in the stroma in relation to the Mg^2+^ dependency of CO_2_ fixation in intact chloroplasts. Biochim. Biophys. Acta 449, 434–446. 10.1016/0005-2728(76)90154-7 11816

[B75] Portis A. R. (1981). Evidence of a low stromal Mg^2+^ concentration in intact chloroplasts in the dark I. Studies with the ionophore A23187. Plant Physiol. 67, 985–989. 10.1104/pp.67.5.985 16661806 PMC425814

[B76] Portis A. R. Chon C. J. Mosbach A. Heldt H. W. (1977). Fructose- and sedoheptulose-bisphosphatase. The site of a possible control of CO_2_ fixation by light-dependent changes of the stromal Mg^2+^ concentration. Biochim. Biophys. Acta 461, 313–325. 10.1016/0005-2728(77)90181-5 19060

[B77] Pottosin I. I. (1992). Single channel recording in the chloroplast envelope. FEBS Lett. 308, 87–90. 10.1016/0014-5793(92)81057-S 1379554

[B78] Pottosin I. I. (1993). One of the chloroplast envelope ion channels is probably related to the mitochondrial VDAC. FEBS Lett. 330, 211–214. 10.1016/0014-5793(93)80275-Y 7689985

[B79] Pottosin I. I. Muñiz J. Shabala S. (2005). Fast-activating channel controls cation fluxes across the native chloroplast envelope. J. Membr. Biol. 204, 145–156. 10.1007/s00232-005-0758-3 16245037

[B80] Pottosin I. I. Schönknecht G. (1995a). Patch clamp study of the voltage-dependent anion channel in the thylakoid membrane. J. Membr. Biol. 148, 143–156. 10.1007/BF00207270 8606363

[B81] Pottosin I. I. Schönknecht G. (1995b). Anion and cation channels in the thylakoid membrane, in Photosynthesis: from Light to Biosphere, Vol. 3, ed Mathis P. (Dorderecht: Kluwer Acad. Publ.), 99–102.

[B82] Pottosin I. I. Schönknecht G. (1996). Ion channel permeable for divalent and monovalent cations in native spinach thylakoid membranes. J. Membr. Biol. 152, 223–233. 10.1007/s002329900100 8672080

[B83] Pottosin I. Shabala S. (2015). Transport across chloroplast membranes: optimizing photosynthesis for adverse environmental conditions. Mol. Plant.. 10.1016/j.molp.2015.10.006. [Epub ahead of print]. 26597501

[B84] Pribil M. Labs M. Leister D. (2014). Structure and dynamics of thylakoids in land plants. J. Exp. Bot. 65, 1955–1972. 10.1093/jxb/eru090 24622954

[B85] Pudelski B. Kraus S. Soll J. Phillipar K. (2010). The plant PRAT proteins- preprotein and amino acid transport in mitochondria and chloroplasts. Plant Biol. 12, 42–55. 10.1111/j.1438-8677.2010.00357.x 20712620

[B86] Pudelski B. Schock A. Hoth S. Radchuk R. Weber H. Hofmann J.. (2012). The plastid outer envelope protein OEP16 affects metabolic fluxes during ABA-controlled seed development and germination. J. Exp. Bot. 63, 1919–1936. 10.1093/jxb/err375 22155670 PMC3295387

[B87] Remiš D. Bulychev A. A. Kurella G. A. (1986). The electrical and chemical components of the protonmotive force in chloroplasts as measured with capillary and pH-sensitive microelecrodes. Biochim. Biophys. Acta 852, 68–73. 10.1016/0005-2728(86)90057-5

[B88] Robinson S. P. (1985). Osmotic adjustment by intact isolated chloroplasts in response to osmotic stress and its effect on photosynthesis and chloroplast volume. Plant Physiol. 79, 996–1002. 10.1104/pp.79.4.996 16664560 PMC1075014

[B89] Röhl T. Motzkus M. Soll J. R. (1999). The outer envelope protein OEP24 from pea chloroplasts can functionally replace the mitochondrial VDAC in yeast. FEBS Lett. 460, 491–494. 10.1016/S0014-5793(99)01399-X 10556523

[B90] Roh M. H. Shingles R. Cleveland M. J. McCarty R. (1998). Direct measurement of calcium transport across chloroplast inner-envelope vesicles. Plant Physiol. 118, 1447–1454. 10.1104/pp.118.4.1447 9847120 PMC34762

[B91] Rolland N. Gurie G. Finazzi J. Kuntz M. Maréchal E. Marringe M.. (2012). The biosynthetic capacities of the plastids and integration between cytoplasmic and chloroplast processes. Annu. Rev. Genet. 46, 233–264. 10.1146/annurev-genet-110410-132544 22934643

[B92] Schindl R. Weghuber J. (2012). “Electrophysiological techniques for mitochondrial channels, patch clamp technique,” ed Kaneez F. S. (InTech), 163–169. Available online at: http://www.intechopen.com/books/patch-clamp-technique/electrophysiological-techniques-for-mitochondrialchannels

[B93] Schönknecht G. Althoff G. Junge W. (1990). The electric unit size of thylakoid membranes. FEBS Lett. 277, 65–68. 10.1016/0014-5793(90)80810-6 1702737

[B94] Schönknecht G. Hedrich R. Junge W. Raschke K. (1988). A voltage-dependent chloride channel in the photosynthetic membrane of a higher plant. Nature 336, 589–592.

[B95] Schönknecht G. Neimanis S. Katona E. Gerst U. Heber U. (1995). Relationship between photosynthetic electron transport and pH gradient across the thylakoid membrane in intact leaves. Proc. Natl. Acad. Sci. U.S.A. 92, 12185–12189. 10.1073/pnas.92.26.12185 11607620 PMC40321

[B96] Schwarz M. Gross A. Steinkamp T. Flügge U. I. Wagner R. (1994). Ion channel properties of the reconstituted chloroplast triose phosphate/ phosphate translocator. J. Biol. Chem. 269, 29481–29489. 7525584

[B97] Shabala L. Ross T. McMeekin T. Shabala S. (2006). Non-invasive microelectrode ion flux measurements to study adaptive responses of microorganisms to the environment. FEMS Microbiol. Rev. 30, 472–486. 10.1111/j.1574-6976.2006.00019.x 16594966

[B98] Shimoni E. Rav-Hon O. Ohad I. Brumfeld V. Reich Z. (2005). Three-dimensional organization of higher-plant chloroplast thylakoid membranes revealed by electron tomography. Plant Cell 17, 2580–2586. 10.1105/tpc.105.035030 16055630 PMC1197436

[B99] Soll J. Bölter B. Wagner R. Hinnah S. C. (2000). …response: the chloroplast outer envelope: a molecular sieve? Trends Plant Sci. 5, 137–138. 10.1016/S1360-1385(00)01579-X 10740293

[B100] Sorgato M. C. Keller B. U. Stühmer W. (1987). Patch-clamping of the inner mitochondrial membrane reveals a voltage-dependent ion channel. Nature 330, 498–500. 10.1038/330498a0 2446143

[B101] Steinkamp T. Hill K. Hinnah S. C. Wagner R. Röhl T. Pohlmeyer K.. (2000). Identification of the pore-forming region of the outer chloroplast envelope protein OEP16. J. Biol. Chem. 275, 11758–11764. 10.1074/jbc.275.16.11758 10766798

[B102] Suchyna T. M. Markin V. S. Sachs F. (2009). Biophysics and structure of the patch and gigaseal. Biophys. J. 97, 738–747. 10.1016/j.bpj.2009.05.018 19651032 PMC2718145

[B103] Szabo I. Zoratti M. (2014). Mitochondrial channels: ion fluxes and more. Physiol. Rev. 94, 519–608. 10.1152/physrev.00021.2013 24692355

[B104] Takizawa K. Cruz J. A. Kanazawa A. Kramer D. M. (2007). The thylakoid proton motive force *in vivo*. Quantitative, non-invasive probes, energetics, and regulatory consequences of light-induced pmf. Biochim. Biophys. Acta 1767, 1233–1244. 10.1016/j.bbabio.2007.07.006 17765199

[B105] Teardo E. Frare E. Segalla A. De Marco V. Giacometti G. M. Szabó I. (2005). Localization of a putative ClC channel in spinach chloroplasts. FEBS Lett. 579, 4991–4996. 10.1016/j.febslet.2005.08.005 16115625

[B106] Teardo E. Segalla A. Formentin E. Zanetti M. Marin O. Giacometti G. M.. (2010). Characterization of a plant glutamate receptor activity. Cell. Physiol. Biochem. 26, 253–262. 10.1159/000320525 20798509

[B107] Tester M. Blatt M. R. (1989). Direct measurement of K^+^ channels in thylakoid membranes by incorporation of vesicles into planar lipid bilayers. Plant Physiol. 91, 249–252. 10.1104/pp.91.1.249 16667005 PMC1061982

[B108] Thaler M. Simonis W. Schönknecht G. (1991). Light-dependent changes of the cytoplasmic H^+^ and Cl^−^ activity in the green alga *Eremosphaera viridis*. Plant Physiol. 99, 103–110. 10.1104/pp.99.1.103 16668835 PMC1080412

[B109] Tikhonov A. N. Khomutov G. B. Ruuge E. K. Blumenfeld L. A. (1981). Electron transport in chloroplasts effects of photosynthetic control monitored by the intrathylakoid pH. Biochim. Biophys. Acta 637, 321–333. 10.1016/0005-2728(81)90171-7

[B110] Tikhonova L. I. Pottosin I. I. Dietz K. J. Schönknecht G. (1997). Fast-activating cation channel in barley mesophyll vacuoles. Inhibition by calcium. Plant J. 11, 1059–1070. 10.1046/j.1365-313X.1997.11051059.x

[B111] Tomaskova Z. Ondrias K. (2010). Mitochondrial chloride channels-What are they for? FEBS Lett. 584, 2085–2092. 10.1016/j.febslet.2010.01.035 20100478

[B112] Ursell T. Agrawal A. Phillips B. (2011). Lipid bilayer mechanics in a pipette with glass-bilayer adhesion. Biophys. J. 101, 1913–1920. 10.1016/j.bpj.2011.08.057 22004745 PMC3192956

[B113] van den Wijngaard P. W. J. Dabney-Smith C. Bruce B. D. Vredenberg W. J. (1999). The mechanism of inactivation of a 50-pS envelope anion channel during chloroplast protein import. Biophys. J. 77, 3156–3162. 10.1016/S0006-3495(99)77146-8 10585937 PMC1300586

[B114] van den Wijngaard P. W. J. Demmers J. A. A. Thompson S. J. Wienk H. L. J. de Kruiff B. Vredenberg W. J. (2000). Further analysis of the involvement of the envelope anion channel PIRAC in chloroplast protein import. Eur. J. Biochem. 267, 3812–3817. 10.1046/j.1432-1327.2000.01419.x 10849000

[B115] van den Wijngaard P. W. J. Vredenberg W. J. (1997). A 50-picosiemens anion channel of the chloroplast envelope is involved in chloroplast protein import. J. Biol. Chem. 272, 29430–29433. 10.1074/jbc.272.47.29430 9367999

[B116] van den Wijngaard P. W. J. Vredenberg W. J. (1999). The envelope anion channel involved in chloroplast protein import is associated with Tic110. J. Biol. Chem. 274, 25201–22520. 10.1074/jbc.274.36.25201 10464239

[B117] van Meer G. Voelker D. R. Feigenson G. W. (2008). Membrane lipids: where they are and how they behave. Nat. Rev. Mol. Cell Biol. 9, 112–124. 10.1038/nrm2330 18216768 PMC2642958

[B118] Viérick A. Rolland N. Joyard J. Ruysschaert J.-M. Homblé F. (2003). Regulation of the anion channel of the chloroplast envelope from spinach. J. Bioenerg. Biomembr. 35, 221–229. 10.1023/A:1024607630952 13678273

[B119] Weber A. P. M. Linka N. (2011). Connecting the plastid: transporters of the plastid envelope and their role in linking plastidial with cytosolic metabolism. Annu. Rev. Plant Biol. 62, 53–77. 10.1146/annurev-arplant-042110-103903 21526967

[B120] Werdan K. Heldt H. W. Milovancev M. (1975). The role of pH in the regulation of carbon fixation in the chloroplast stroma. Studies on CO_2_ fixation in the light and dark. Biochim. Biophys. Acta. 396, 276–292. 10.1016/0005-2728(75)90041-9 239746

[B121] Wolosiuk R. A. Hertig C. M. Nishizawa A. N. Buchanan B. B. (1982). Enzyme regulation in C-4 photosynthesis. 3. Role of Ca^2+^ in thioredoxin-linked activation of sedoheptulose bisphosphatase from corn leaves. FEBS Lett. 140, 31–35. 10.1016/0014-5793(82)80514-0

[B122] Wu W. Berkowitz G. A. (1992). Stromal pH and photosynthesis are affected by electroneutral K^+^ and H^+^ exchange through chloroplast envelope ion channels. Plant Physiol. 98, 666–672. 10.1104/pp.98.2.666 16668693 PMC1080242

[B123] Wu W. Peters J. S. Berkowitz G. A. (1991). Surface-charge mediated effects of Mg^2^+ on K^+^ flux across the chloroplast envelope are associated with regulation of stromal pH and photosynthesis. Plant Physiol. 97, 580–587. 10.1104/pp.97.2.580 16668438 PMC1081046

[B124] Zifarelli G. Pusch M. (2010a). CLC transport proteins in plants. FEBS Lett. 584, 2122–2127. 10.1016/j.febslet.2009.12.042 20036660

[B125] Zifarelli G. Pusch M. (2010b). The role of protons in fast and slow gating of the *Torpedo* chloride channel ClC-0. Eur. Biophys. J. 39, 869–875. 10.1007/s00249-008-0393-x 19132363

[B126] Zifarelli G. Pusch M. (2012). A kick-start for CLC antiporters' pharmacology. Chem. Biol. 19, 1358–1359. 10.1016/j.chembiol.2012.10.009 23177190

